# Corrosion Resistance of Mg_72_Zn_24_Ca_4_ and Zn_87_Mg_9_Ca_4_ Alloys for Application in Medicine

**DOI:** 10.3390/ma13163515

**Published:** 2020-08-09

**Authors:** Andrzej Fijołek, Janusz Lelito, Halina Krawiec, Jagoda Ryba, Łukasz Rogal

**Affiliations:** 1 Faculty of Foundry Engineering, AGH University of Science and Technology, Al. A. Mickiewicza 30, 30-059 Kraków, Poland; and.fijolek@gmail.com (A.F.); jagoda.ryba1990@gmail.com (J.R.); 2 Institute of Metallurgy and Materials Science of Polish Academy of Sciences in Cracow, 25 Reymonta Street, 30-059 Kraków, Poland; l.rogal@imim.pl

**Keywords:** biodegradability, corrosion, Mg-Zn-Ca alloy, Zn-Mg-Ca alloy, ringer solution

## Abstract

The aim of this work was to monitor the corrosion rate of the Mg_72_Zn_24_Ca_4_ and Zn_87_Mg_9_Ca_4_ alloys. The purity of the alloying elements was 99.9%. The melt process was carried out in an induction furnace. The melting process took place under the cover of an inert gas (argon). The copper form was flooded by liquid alloy. Then, in order to obtain ribbons, the cast alloy, in rod shape, was re-melted on the melt spinning machine. The corrosion resistance of both alloys has been determined on the basis of the following experiments: measurements of the evolution of OCP (open circuit potential), LSV (linear sweep voltamperometry) and EIS (electrochemical impedance spectroscopy). All corrosion tests were carried out in Ringer’s solution at 37 °C and pH 7.2. The corrosion tests have revealed that the zinc alloy, Zn_87_Mg_9_Ca_4,_ exhibits significantly higher corrosion resistance in the Ringer solution compared to the magnesium alloy, Mg_72_Zn_24_Ca_4_. Moreover, it has been shown that the cathodic reaction proceeds faster on the surface of ribbons. EIS measurements show that the dissolution of Mg alloy proceeds with two steps: transfer of Mg^2+^ ions to the Ringer solution and then the formation of the corrosion products, which are deposited on the surface of magnesium alloy. It has been revealed, too, that for both bulk materials, diffusion of chloride ions through the corrosion product’s layer takes place.

## 1. Introduction

Biodegradable metals in recent years have attracted considerable attention as potential orthopedic implants. Mg and its alloys [1,2,3,4,5] including Mg-Ca [6], Mg-RE [7,8], Mg-Sr [9], Mg-Zn [10,11] and Mg-based bulk metallic glasses (BMGs) [12,13] are considered biodegradable metallic materials.

Due to very good biodegradability, biocompatibility and non-toxicity, magnesium and its alloys are widely used in the biomedical sector [14]. Large amounts of Mg^2+^ are present in the human body. These ions participate in many metabolic reactions, biological mechanisms, and their excess is easily excreted via urine [15].

The human body has an inborn tolerance for the absorption of magnesium, zinc and calcium. The daily intake ordered for these elements is 1000 mg day^−1^ of Ca [16], 420 mg day^−1^ of Mg [17], 10 mg day^−1^ of Zn [18]. In addition, the presence of these ions in the human body is associated with antibacterial action against bacteria, such as: *Escherichia coli*, *Pseudomonas aeruginosa*, *Staphylococcus aureus*, and against the occurrence of anaerobes in the oral cavity [19,20].

Magnesium alloys have a complex microstructure, which consists of matrix, intermetallic phases, eutectic, and precipitates. Moreover, the alloying elements are not uniformly distributed within the magnesium matrix. Therefore, the corrosion of magnesium alloys is not uniform. The corrosion process usually starts at the interface of matrix/intermetallic phases. The intermetallic phases can behave as cathodic or anodic places depending on their chemical composition. Type, size and distribution of precipitates within the magnesium matrix have a significant influence on the corrosion resistance of Mg alloys [21,22,23,24]. It was found that good corrosion resistance of Mg alloys can be achieved when structural uniformity is present. The amorphous magnesium-based alloys have a lower corrosion rate in comparison to crystalline specimens, which have precipitates in their microstructure [25,26]. The amorphous structure ensures greater ability of passivation and lower susceptibility to local corrosion of magnesium-based alloys.

Recently, in the literature [5,14,15,27,28,29,30], there is considerable interest in zinc and its alloys as a material for orthopedic implants and devices for cardiovascular interventional. The interest in this material is related, on the one hand, to their biodegradability and, on the other hand, to very good biocompatibility. Additionally, it should be noted that zinc plays a key role in the human body. It takes part in the metabolism of the human body, for example in the composition of essential enzymes for the synthesis of proteins and transcription factors [30,31,32]. It should be noted that the released zinc from the implants during its dissolution is non-toxic and does not cause side effects. The tests performed on mice showed good zinc biocompatibility in the abdominal aorta and the femur of the animal. It causes strong adherence to the wire of healthy arterial tissue [30,31] and the formation of new bone around the pins [5,27]. Additionally, the electrochemical potential of zinc is located between the value of the potentials measured for magnesium and iron. At the same time, the biodegradable use of metal implants is still being questioned. This is especially the case with implants made of alloys based on magnesium or iron [16,32,33,34,35,36]. On the one hand, it is related to the too fast degradation of implants based on magnesium alloys, caused by rapid corrosion, preventing effective bone fusion, and on the other hand, too slow degradation of implants based on iron alloys, resulting in the appearance of permanent implants [27,32,36]. Despite the great advantages of zinc, the use of pure zinc as a material for biodegradable implants is limited due to low mechanical properties such as strength, plasticity and hardness.

Calcium is a major component in human bone and is essential in chemical signaling with cells [33,34]. In addition, magnesium is necessary to incorporate calcium into the bone [30,37], which might be expected to be beneficial to the bone healing with the co-releasing of Mg and Ca ions. Calcium is also beneficial to the grain refinement of magnesium alloys. The solubility limit of Ca in Mg is 1.34 wt % [30,38]. The Mg-Ca alloys are mainly composed of α (Mg) phase and Mg_2_Ca phase [30,39]. With increasing Ca content, more and coarser Mg_2_Ca phase precipitates along grain boundaries, weakening both the mechanical property and corrosion resistance of as-cast Mg-Ca alloy [30,39,40].

In this work, calcium was selected as the alloying component to develop ternary magnesium–zinc–calcium and zinc–magnesium–calcium alloys. The corrosion resistance of both alloys in the Ringer solution has been investigated.

## 2. Materials and Methods

In order to obtain the Mg_72_Zn_24_Ca_4_ and Zn_87_Mg_9_Ca_4_ alloys, alloying elements with a purity of 99.99% were used. Then, the melt was carried out in a resistance furnace under argon as an inert gas. Afterwards, the Mg_72_Zn_24_Ca_4_ and Zn_87_Mg_9_Ca_4_ alloys were cast into the steel metal mold to obtain a cylinder sample with the following dimensions: diameter = 20 mm and height = 50 mm. Final dimensions of samples for further testing after machining: diameter = 10 mm and height = 50 mm. Then, in order to obtain ribbons, the cast alloy, in rod shape, was re-melted on the melt spinning machine. The average thickness of the ribbons, produced in the melt spinning process, was approximately between 100–150 µm. The rotational speed of the wheel during the melt spinning of ribbons was 35 m/min. Then, the corrosion resistance of bulk Mg_72_Zn_24_Ca_4_ and Zn_87_Mg_9_Ca_4_ alloys has been determined.

The corrosion tests have been performed in the Ringer solution at 37 °C. The chemical composition of the Ringer solution is given in Table 1.

The bulk material of Mg_72_Zn_24_Ca_4_ and Zn_87_Mg_9_Ca_4_ alloys used for corrosion tests were ground on abrasive papers with gradations from 1200 to 4000. After each polishing step, the samples were cleaned for 5 min in ethanol using ultrasound.

In order to determine the corrosion resistance of the Mg_72_Zn_24_Ca_4_ and Zn_87_Mg_9_Ca_4_ alloys evolution of corrosion potential versus time (OCP—open circuit potential), potentiodynamic polarization curves (LSV—linear sweep voltamperometry) were measured. The potential scan rate for linear sweep voltamperometry was 1 mV/s. Afterwards, electrochemical impedance spectroscopy (EIS) was performed. EIS spectra were measured at the OCP, the amplitude of the perturbation signal was 10 mV, and the frequency range was from 100 kHz to 0.003 Hz. Experimental EIS data were analyzed by being fit to an equivalent electrical circuit model by using Zview software. In order to obtain the steady state, the specimens were immersed in the Ringer solution for 24 h and then the EIS spectra were measured for bulk material samples (before melt spinning process). All electrochemical measurements were performed in the aerated Ringer’s solution at 37 °C. The corrosion tests were performed by using the AUTOLAB PGSTAT 128 potentiostat.

The microstructure of Mg_72_Zn_24_Ca_4_ and Zn_87_Mg_9_Ca_4_ alloys were investigated by using a scanning electron microscopy SEM/EDS (JEOL JSM-5500LV, Tokyo, Japan). In order to determine the intermetallic phases present in these alloys, XRD measurements were performed using Philips PW 1840 X-ray diffractometer (Cambridge, UK) with Co K α radiation (λ = 1.78896A) with the X’Pert system, equipped with the ATC-3 texture goniometer using Philips APD company programs and ICDD (the International Centre For Diffraction Data) crystallographic database.

## 3. Results

**Figure 1:**
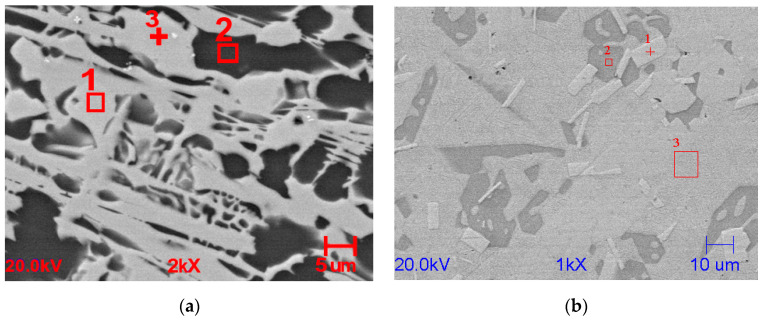
SEM/EDS images revealed after mechanical polishing for Mg_72_Zn_24_Ca_4_ (**a**) and Zn_87_Mg_9_Ca_4_ (**b**) alloys, respectively.

Figure 1 depicts SEM/EDS images for Mg_72_Zn_24_Ca_4_ (a) and for Zn_87_Mg_9_Ca_4_ (b) alloys, respectively. The chemical composition of individual phases was determined by EDS (secondary electron mode) analysis and is given in Table 2 and Table 3.

As shown in Figure 1, the microstructure of the as-cast alloy consists of the α-Mg matrix, particle phases, and skeleton phases [41,42,43,44]. The phases occurring in the tested alloys are Mg_39_Zn for the Mg_72_Zn_24_Ca_4_ alloy (Figure 1a) and Mg_2_Zn_11_ for the Zn_87_Mg_9_Ca_4_ alloy (Figure 1b). Microstructure consists of primary dendrites of Zn (white areas) and Zn + Mg_2_Zn_11_ eutectic mixture (dark areas) in the case of Zn alloy, Figure 1b. It has been shown, that the volume fraction of eutectic mixture is increased with higher content of Zn.

**Figure 2:**
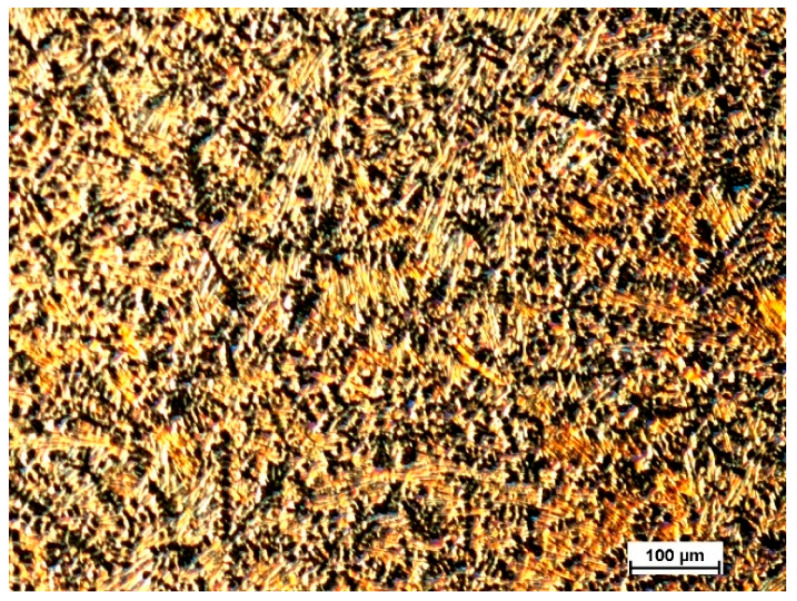
Light microscope image of microstructure revealed after etching in 2% nital solution for Mg_72_Zn_24_Ca_4_ bulk material sample—before the melt spinning process.

**Figure 3:**
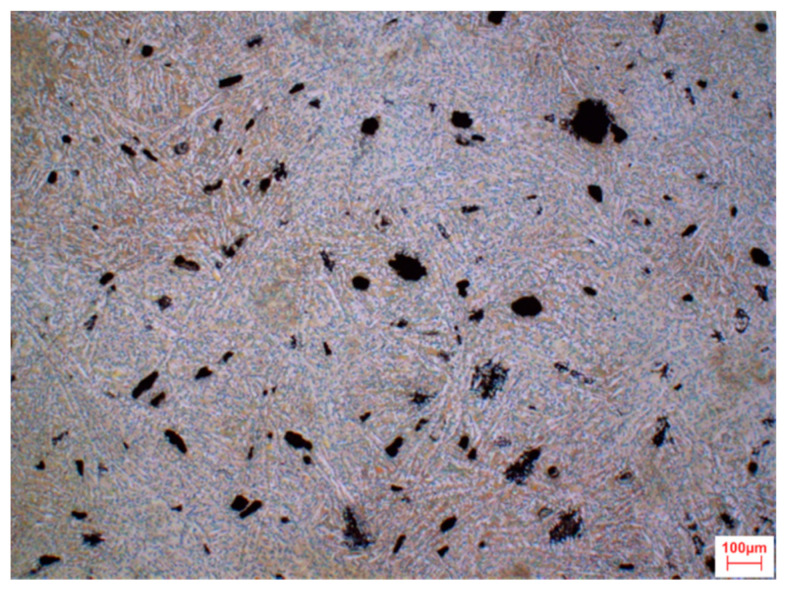
Light microscope image of microstructure revealed after etching in 2% nital solution for Zn_87_Mg_9_Ca_4_ bulk material sample—before the melt spinning process.

Figure 2 and Figure 3 depicts the microstructure image for the Mg_72_Zn_24_Ca_4_ bulk and Zn_87_Mg_9_Ca_4_ material sample—before the melt spinning process.

**Figure 4:**
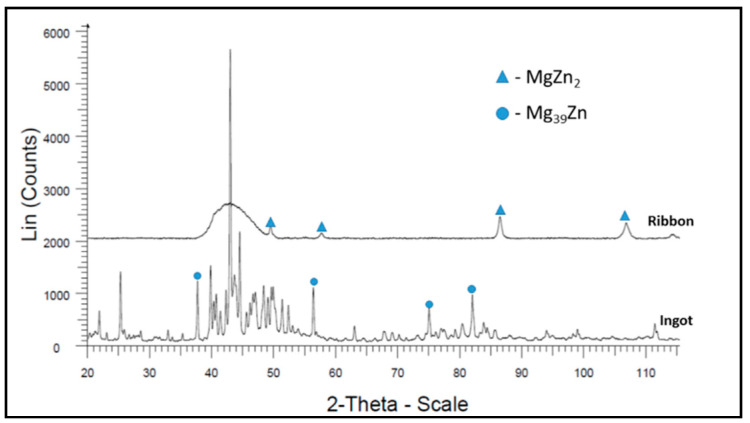
X-ray diffraction results for Mg_72_Zn_24_Ca_4_ alloy samples.

The XRD results for Mg_72_Zn_24_Ca_4_ alloy ingot samples (before the melt spinning process) and ribbon (after the melt spinning process) are presented in Figure 4.

**Figure 5:**
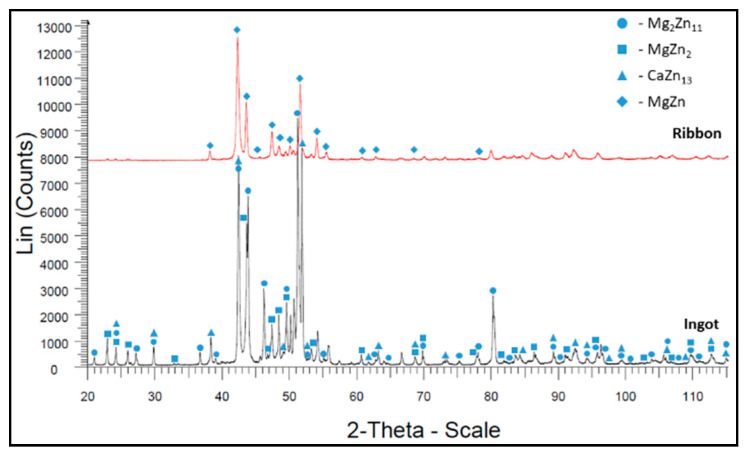
X-ray diffraction results for Zn_87_Mg_9_Ca_4_ alloy samples.

The XRD results for Zn_87_Mg_9_Ca_4_ alloy ingot samples (before the melt spinning process) and ribbon (after the melt spinning process) are presented in Figure 5.

X-ray diffraction (XRD) analysis was carried out to verify the glassy nature of the ribbons after the melt spinning process (Figure 4 and Figure 5). Figure 4 shows the results of XRD tests for both the ribbon and the ingot of the Mg_72_Zn_24_Ca_4_ alloy. The ribbon has an amorphous structure with a small amount of the MgZn_2_ intermetallic phase, while the ingot is characterized by a crystalline structure [45,46]. Figure 5 presents MgZn and CaZn_13_ phase for the ribbon of Zn_87_Mg_9_Ca_4_ alloy after the melt spinning process. This fact addresses the large fragmentation of the structure that followed the melt spinning process. It indicates that it is not an amorphous structure as in the case of the Mg_72_Zn_24_Ca_4_ alloy ribbon. In addition, the Zn_87_Mg_9_Ca_4_ alloy strip shown in Figure 5 has more phase spots (peaks of MgZn and CaZn_13_ phase) after X-ray diffraction compared to the Mg_72_Zn_24_Ca_4_ alloy ribbon shown in Figure 4.

**Figure 6:**
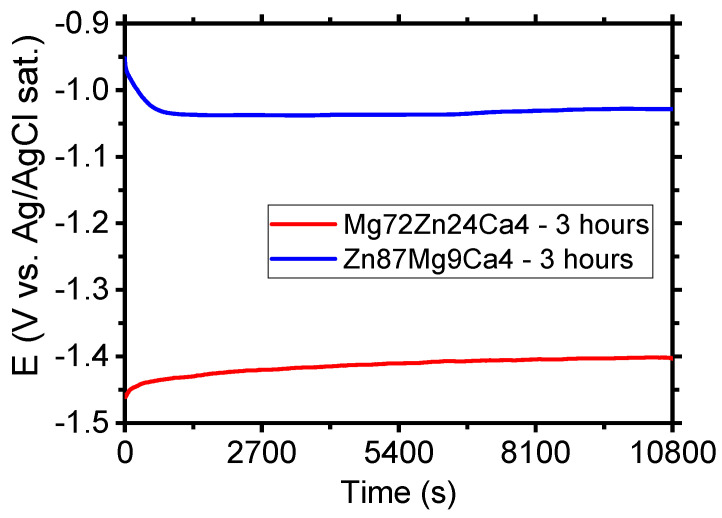
Evolution of open circuit potential (OCP) for Mg_72_Zn_24_Ca_4_ and Zn_87_Mg_9_Ca_4_ alloys specimens in Ringer’s solution at 37 °C; pH 7.2 after 3 h.

Figure 6 shows the evolution of open circuit potential (OCP) for Mg_72_Zn_24_Ca_4_ and Zn_87_Mg_9_Ca_4_ specimens in the Ringer solution. After three hours of immersion of specimens in the Ringer solution, the following values of OCP were measured: −1.40 V and −1.03 V for Mg_72_Zn_24_Ca_4_ and Zn_87_Mg_9_Ca_4_ specimens, respectively. It can be noticed that after 2700 s, the OCP reached the stable value for both alloys. This means that the steady state was attained. The OCP measured for Zn_87_Mg_9_Ca_4_ alloy is 375 mV higher than the one measured for the Mg_72_Zn_24_Ca_4_ alloy.

**Figure 7:**
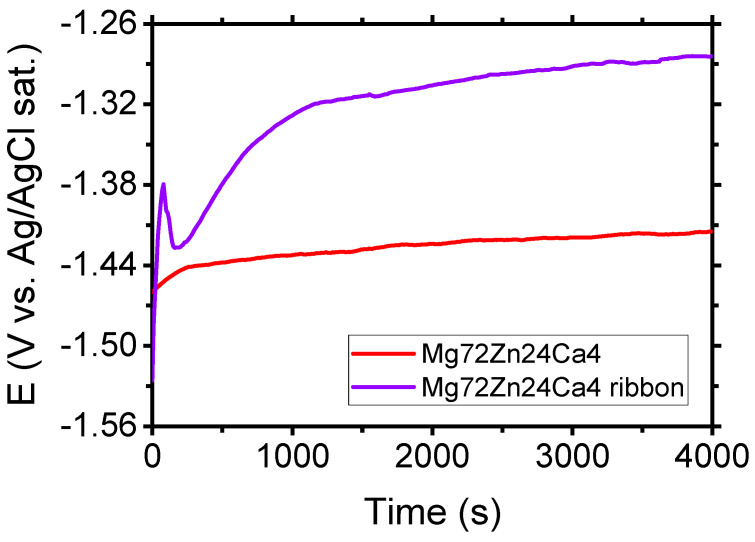
Evolution of OCP for Mg_72_Zn_24_Ca_4_ bulk material and Mg_72_Zn_24_Ca_4_ ribbon specimens in Ringer’s solution at 37 °C; pH 7.2 after four thousand seconds.

Figure 7 shows the evolution of open circuit potential (OCP) for the Mg_72_Zn_24_Ca_4_ bulk material sample (before the melt spinning process) and Mg_72_Zn_24_Ca_4_ ribbon (after the melt spinning process) specimens in the Ringer solution. After four thousand seconds of immersion of specimens in the Ringer solution, the following values of OCP were measured: −1.417 V and −1.284 V for Mg_72_Zn_24_Ca_4_ bulk material sample and Mg_72_Zn_24_Ca_4_ for ribbon specimens, respectively. The OCP measured for Mg_72_Zn_24_Ca_4_ ribbon is 133 mV higher than the one measured for the Mg_72_Zn_24_Ca_4_ bulk material sample.

**Figure 8:**
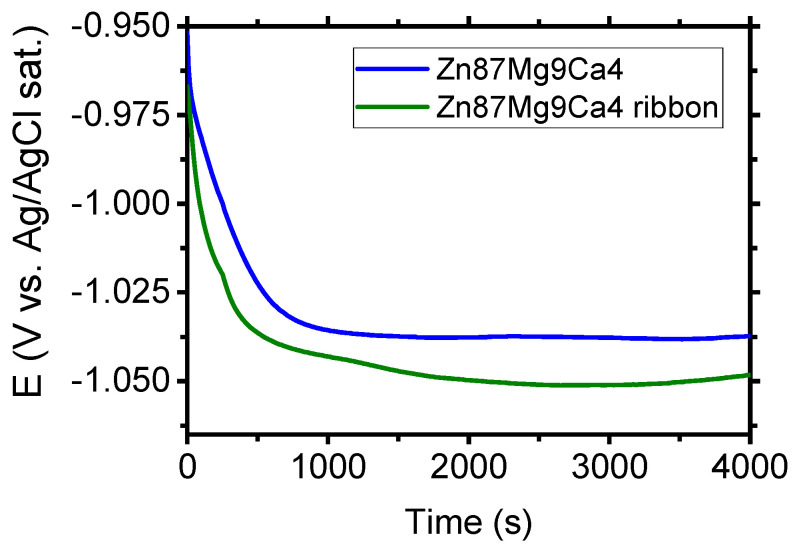
Evolution of OCP for Zn_87_Mg_9_Ca_4_ bulk material and Zn_87_Mg_9_Ca_4_ ribbon specimens in Ringer’s solution at 37 °C; pH 7.2 after four thousand seconds.

Figure 8 depicts the evolution of open circuit potential (OCP) for the Zn_87_Mg_9_Ca_4_ bulk material sample (before the melt spinning process) and Zn_87_Mg_9_Ca_4_ ribbon (after the melt spinning process) specimens in the Ringer solution. After four thousand seconds of immersion of specimens in the Ringer solution, the following values of OCP were measured: −1.048 V and −1.037 V for Mg_72_Zn_24_Ca_4_ bulk material sample and Mg_72_Zn_24_Ca_4_ for ribbon specimens, respectively. The OCP measured for the Zn_87_Mg_9_Ca_4_ bulk material sample is 11 mV higher than the one measured for the Zn_87_Mg_9_Ca_4_ ribbon.

The increase in corrosion potential versus time can cause: the formation of the passive film, the decrease in anodic reaction rate, or the increase in the cathodic reaction rate. The increase in cathodic reaction is related with the increase in oxygen concentration dissolved in the solution [47].

**Figure 9:**
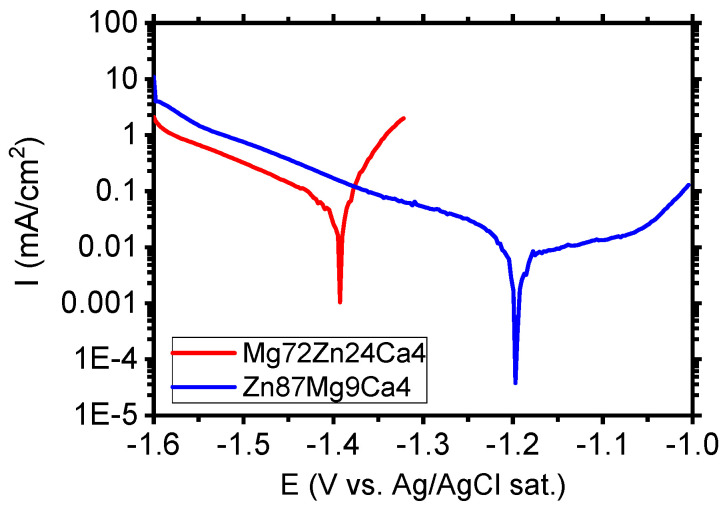
Polarization curves performed for Mg_72_Zn_24_Ca_4_ and Zn_87_Mg_9_Ca_4_ alloys in Ringer’s solution at 37 °C, pH 7.2.

Figure 9 depicts the polarization curves determined for Mg_72_Zn_24_Ca_4_ and Zn_87_Mg_9_Ca_4_ in the Ringer solution. The Mg_72_Zn_24_Ca_4_ exhibits the active corrosion. In the anodic branch, the sharp increase in the current density is observed. The increase in the anodic current density is related with the dissolution of magnesium according with the reaction (1).

(1) $$\mathrm{Mg}\;\to {\mathrm{Mg}}^{2+}+2e$$

During the cathodic process, the hydrogen is evolved, and the hydroxide ions are formed, as shown in reaction (2).

(2) $$2H_{2}O+2e^{-}\;\to H_{2}+2OH^{-}$$

The reaction of Mg^2+^ ions with the OH^-^ ions cause the formation of corrosion product Mg(OH)_2_. The corrosion products, which contain mainly Mg(OH)_2_, are porous and do not protect the surface of alloy against corrosion. The current density registered in the cathodic branch for Zn_87_Mg_9_Ca_4_ (blue curve) is higher than the one measured for Mg alloy (red curve) in Figure 9. This suggests that the oxygen reduction (cathodic reaction) is promoted on the surface of the Zn_87_Mg_9_Ca_4_ alloy. In the anodic domain, the current plateau is visible in the potential range between −1.2 V and −1.05 V. The current density in the plateau is 11 µA/cm^2^. This result indicates that the Zn_87_Mg_9_Ca_4_ alloy undergoes the passivation in the Ringer solution. At potential values higher than −1.0 V, the passive film undergoes the anodic dissolution and corrosion of the Zn_87_Mg_9_Ca_4_ alloy.

The corrosion tests performed in the Ringer solution have revealed that the zinc alloy, Zn_87_Mg_9_Ca_4,_ exhibits higher corrosion resistance than the Mg_72_Zn_24_Ca_4_ alloy.

**Figure 10:**
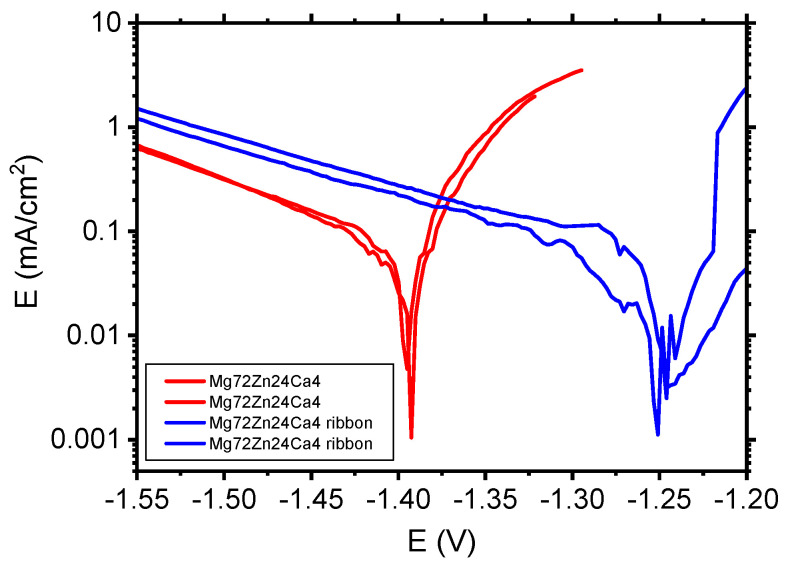
Polarization curves performed for Mg_72_Zn_24_Ca_4_ bulk material samples and Mg_72_Zn_24_Ca_4_ ribbons in Ringer’s solution at 37 °C, pH 7.2.

Figure 10 depicts the polarization curves determined for Mg_72_Zn_24_Ca_4_ bulk material samples (red curves) and Mg_72_Zn_24_Ca_4_ ribbons (violet curves) in the Ringer solution.

The corrosion tests in Figure 10 performed in the Ringer solution have revealed that the Mg_72_Zn_24_Ca_4_ ribbons (blue curves) exhibit higher corrosion resistance than the Mg_72_Zn_24_Ca_4_ bulk material specimens (red curves). The cathodic current density of ribbon is higher compared to the current density registered for bulk material Mg_72_Zn_24_Ca_4_. Such results confirm that the cathodic reaction (HER) proceeds preferentially on ribbon surface. This causes the shift of the corrosion potential of about 150 mV in a more anodic direction. XRD measurements have revealed that the ribbon produced on the base of Mg_72_Zn_24_Ca_4_ has an amorphous structure. The amorphous structure determined better corrosion resistance of ribbons in the Ringer solution.

**Figure 11:**
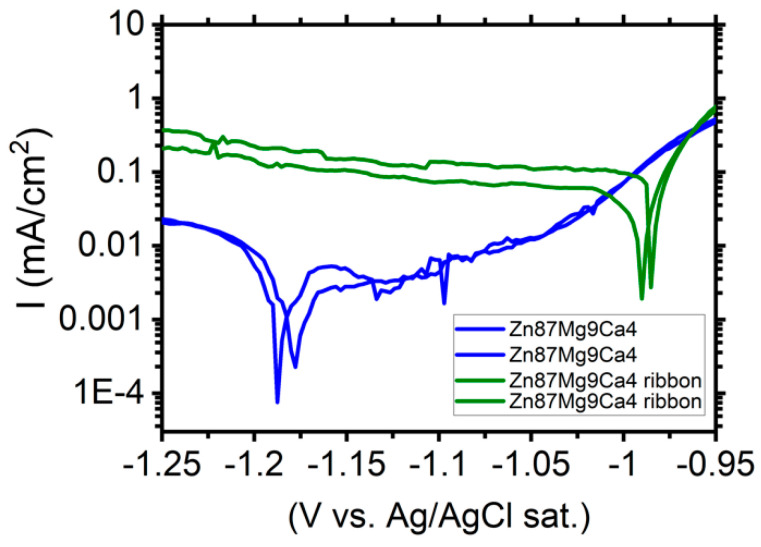
Polarization curves performed for Zn_87_Mg_9_Ca_4_ bulk material samples (blue curves) and Zn_87_Mg_9_Ca_4_ ribbons (olive curves) in Ringer’s solution at 37 °C, pH 7.2.

Figure 11 shows the polarization curves determined for Zn_87_Mg_9_Ca_4_ bulk material samples (blue curves) and Zn_87_Mg_9_Ca_4_ ribbons (olive curves) in the Ringer solution.

Cathodic current density registered for the ribbon surface is one order of magnitude higher compared to the one measured for the bulk material Zn_87_Mg_9_Ca. Therefore, the cathodic reaction (HER) proceeds preferentially on the surface of ribbons. XRD measurements (Figure 5) have revealed that the ribbons produced on the base of Zn_87_Mg_9_Ca_4_ are not totally amorphous. When the corrosion potential was reached, the anodic current density sharply increases and no passive range was detected (olive curves, Figure 11). The corrosion tests, such as the evolution of OCP (Figure 8) and LSV (Figure 11), indicate that the Zn_87_Mg_9_Ca4 ribbon does not reveal much better corrosion resistance than the bulk alloy. This behavior is related to the partial crystalline structure of ribbons.

**Figure 12:**
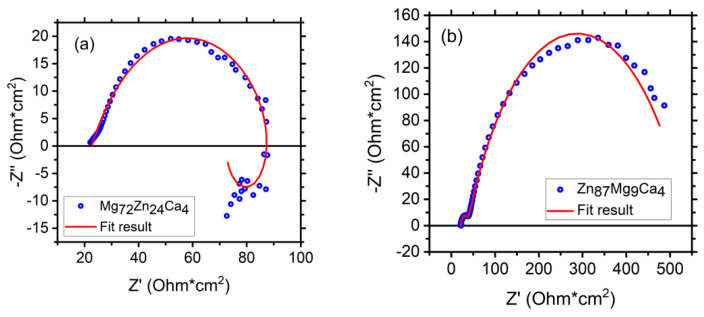
Polarization curves performed for the Zn_87_Mg_9_Ca_4_ bulk material samples (blue curves) and Zn_87_Mg_9_Ca_4_ ribbons (olive curves) in Ringer’s solution at 37 °C, pH 7.2.

Figure 12 depicts fitting results of EIS measurements (Nyquist diagrams) for Mg_72_Zn_24_Ca_4_ (a) and Zn_87_Mg_9_Ca_4_ (b) bulk material samples—before the melt spinning process. The EIS spectra were obtained after 24 h of immersion of both alloys in Ringer’s solution at 37 °C, pH = 7.2.

**Figure 13:**
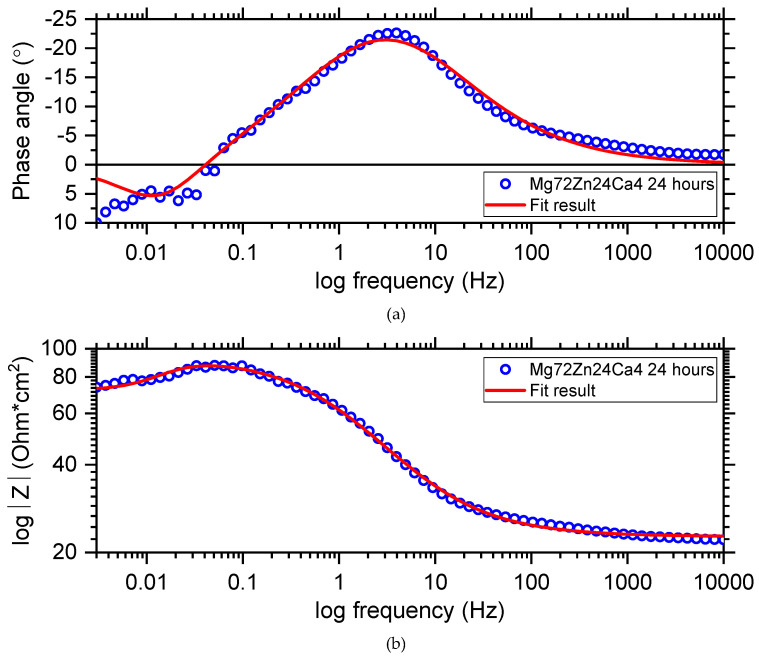
Bode diagrams (**a**,**b**) with respective fitting line for the Mg_72_Zn_24_Ca_4_ alloy sample of immersion at open circuit potential in Ringer’s solution at 37 °C.

**Figure 14:**
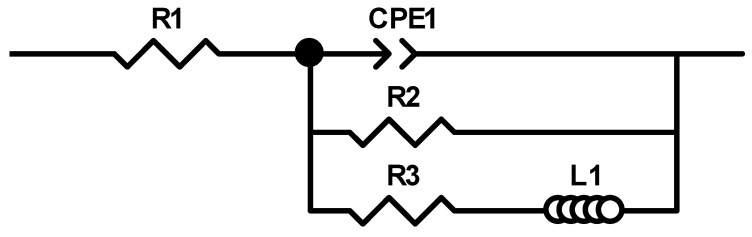
The equivalent circuit used for fitting the experimental electrochemical impedance spectroscopy (EIS) Mg_72_Zn_24_Ca_4_ sample immersed in Ringer’s solution at 37 °C.

Figure 13 depicts the electrochemical impedance spectrum performed for the Mg_72_Zn_24_Ca_4_ alloy in the Ringer solution at 37 °C and pH 7.2. The EIS spectrum revealed the presence of one capacitive loop at the high frequency range and one inductive loop at the low frequency range. The electrical equivalent circuit (EEC), used to fit the EIS diagram for the Mg_72_Zn_24_Ca_4_ alloy, is shown in Figure 14. The red line represents the results obtained from the fitting of EEC (Figure 14). Due to the deformation of Bode’s diagram in the center (Figure 13), a constant phase element (CPE1) was introduced instead of the capacitor. The (R1) represents the solution resistance and (R2) the charge transfer resistance (the resistance to the electron transfer of the faradic process), respectively. The (CPE1) is a constant phase element related to the surface film resistance, (R3) is surface layer resistance and induction, (L) is related to the adsorption of corrosion products on the surface of the Mg_72_Zn_24_Ca_4_ alloy [48,49,50]. The impedance of constant phase element (CPE) is defined by the following Equation (3) [51]:

(3) $${\hat{Z}}_{\mathrm{CPE}}=\frac{1}{Z{\left(j\omega \right)}^{\phi }},$$

where:

CPE-T—the capacitance parameter in F∙cm^−2^∙s^ϕ−1^ which depends on the electrode potential.

Φ—is related to the angle of rotation of the pure capacitive line on the complex plane plots: α = 90°(1-ϕ).

When ϕ = 1, Equation (3) represents pure capacitance for infinite Warburg impedance that is obtained for ϕ = 0.5, pure resistance for ϕ = 0, and pure inductance for ϕ = −1.

The fitting parameters obtained from the EIS measurement performed for the Mg_72_Zn_24_Ca_4_ alloy are presented in Table 4.

Dissolution of the magnesium alloy, according to reaction (1), causes the formation Mg^2+^ ions and the electrons are transferred through interface metal/electrolyte. The magnesium ions react with the OH^-^ ions and the surface layer consists mainly of the Mg(OH)_2_ that forms. The corrosion products are deposited on the surface of Mg_72_Zn_24_Ca_4_. Therefore, the inductive loop is visible at the low frequency in the EIS spectrum (Figure 13). The value of CPE1-P equals 0.65, which indicates that the diffusion process proceeds through the surface layer formed on the Mg_72_Zn_24_Ca_4_ alloy. This result indicates that the surface layer (corrosion products) formed on the Mg alloy does not have the capacitive properties.

**Figure 15:**
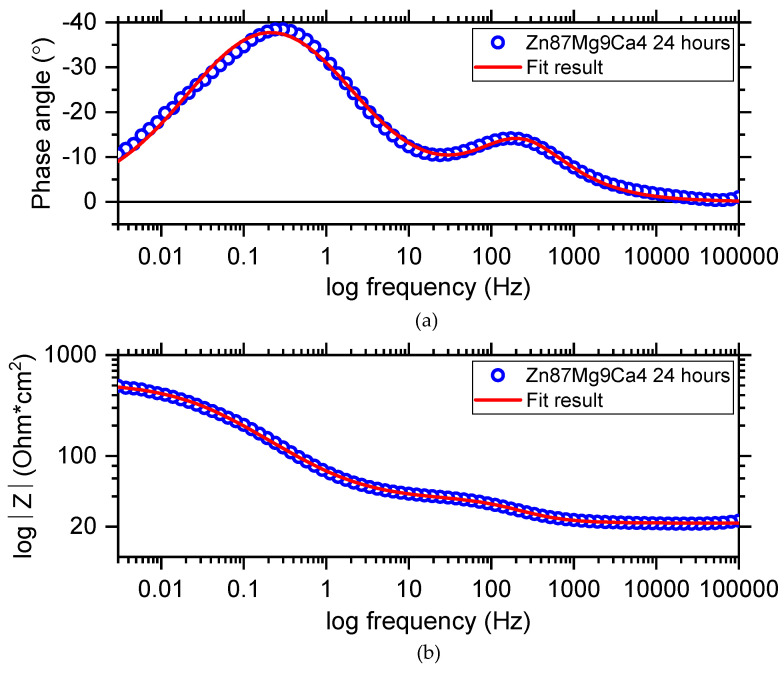
Bode diagram (**a**,**b**) obtained for the Zn_87_Mg_9_Ca_4_ alloy sample immersed at open circuit potential in Ringer’s solution at 37 °C for 24 h.

**Figure 16:**
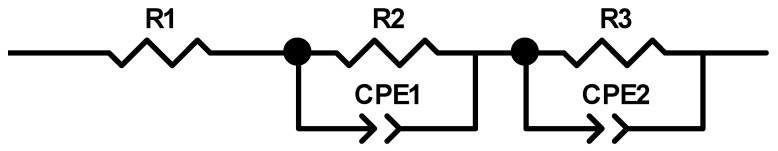
Bode diagram (**a**,**b**) obtained for the Zn_87_Mg_9_Ca_4_ alloy sample immersed at open circuit potential in Ringer’s solution at 37 °C for 24 h.

Figure 15 shows the Bode diagram determined for the Zn_87_Mg_9_Ca_4_ alloy specimen immersed at open circuit potential in Ringer’s solution at 37 °C for 24 h. Blue circles represent the experimental points and the red line represents the results obtained from fitting the electrical equivalent circuit (EEC). The electrical equivalent circuit (EEC) used to fit the EIS diagram obtained for the Zn_87_Mg_9_Ca_4_ alloy is shown in Figure 16. The circuit for this alloy consists of the following elements: R1—solution resistance, R2—change transfer resistance and R3—resistance of the surface layer, CPE1—constant phase element related to the double layer capacity, CPE2—constant phase element related to the surface layer formed at the surface of the Zn_87_Mg_9_Ca_4_ alloy. On the Bode diagram, two capacitive loops are visible, one at the high frequency (HF) and the second at the low frequency (LF).

The change transfer reaction is related to the dissolution of Zn alloys into electrolytes according to reaction (4).

(4) $$\mathrm{Zn}\;\to \;Zn^{2+}+2e,$$

During the cathodic process (HER, reaction (2)), hydroxide ions are produced, and then they react with the Zn^2+^ ions and a zinc hydroxide layer is formed at the surface of the Zn_87_Mg_9_Ca_4_ alloy; reaction (5).

(5) $$Zn^{2+}+2OH^{-}\;\to \;\mathrm{Zn}{\left(\mathrm{OH}\right)}_{2},$$

The presence of the high concentration of Cl^−^ ions in the Ringer solution causes their diffusion through the surface layer, and converts Zn(OH)_2_ to more soluble chloride salts Zn_5_(OH)_8_Cl_2_. The CPE2-P values equal 0.67 (Table 5), confirming that the diffusion of Cl^-^ ions through the hydroxides layer can take place.

In order to summarize, the obtained results clearly show that the bulk material, Zn_87_Mg_9_Ca_4,_ exhibits higher corrosion resistance than the Mg_72_Zn_24_Ca_4_ alloy. Both alloys contain the same amount of calcium. Notably, the ratio of zinc to magnesium is different, being 3/1 and 9/1 for Mg_72_Zn_24_Ca_4_ and Zn_87_Mg_9_Ca_4_ alloys, respectively. The higher ratio of zinc to magnesium results in the cathodic and anodic processes. The higher current density in the cathodic branch is measured for Zn_87_Mg_9_Ca_4_ alloy. In turn, in the anodic branch, the passive region is visible for the Zn_87_Mg_9_Ca_4_ alloy. The high ratio of zinc to magnesium causes the formation of the passive film on its surface, which consists mainly of zinc oxide and zinc hydroxides. Comparison of corrosion behavior of bulk and ribbon materials exhibits the significant difference between them, especially in the cathodic area. The cathodic current density was higher for ribbons of both alloys. This effect is significantly noticeable especially for Zn_87_Mg_9_Ca_4_ alloy where the cathodic current density is one order of magnitude higher compared to the bulk material (Figure 11). XRD measurements have revealed that the ribbon obtained for the Zn_87_Mg_9_Ca_4_ alloy has a partially crystalline microstructure. During the melt spinning process, the cooling rate is very fast which causes the formation of a refine microstructure. The presence of numerous grain boundaries promotes the cathodic reduction reaction (HER) and hinders the passivation process, as is shown in Figure 11 (olive curves). Similar to the results obtained by other researchers [25,26], these results confirm that the amorphous microstructure has a beneficial effect on the corrosion resistance of magnesium-based alloys in Ringer’s solution.

## 4. Conclusions

The corrosion tests have revealed that the Mg_72_Zn_24_Ca_4_ alloy undergoes the active corrosion in the Ringer solution. However, the Zn_87_Mg_9_Ca_4_ alloy undergoes the passivation but at potential values higher than −1.05 E (V vs. Ag/AgCl) (3M KCl), hence, the active dissolution of Zn alloy proceeds. It should be noticed that the cathodic reaction (HER) is favored at the surface of the Zn87Mg9Ca4 alloy. The corrosion of the Mg_72_Zn_24_Ca_4_ alloy in the Ringer solution is related to the dissolution of Mg, and then, to the adsorption of the corrosion product, Mg(OH)_2,_ on its surface. Degradation of the Zn_87_Mg_9_Ca_4_ alloy in the Ringer solution consists of two consecutive steps: (1) formation of the surface layer, (2) diffusion of the chloride ions through the surface layer.

The corrosion tests have revealed that the Zn_87_Mg_9_Ca_4_ alloy (bulk material) exhibits significantly higher corrosion resistance than the Mg_72_Zn_24_Ca_4_ alloy (bulk material) in the Ringer solution. The results obtained in this study has revealed that if the ribbons are amorphous, they exhibit better corrosion resistance than bulk material samples. This fact is confirmed by the observed higher cathodic current density for ribbons and their longer cathodic branch in relation to the bulk material samples.

This result indicates that the cathodic reaction proceeds faster on the surface of the ribbon.

The metallic materials, which are potential candidates for biomedical application, should be nontoxic and their corrosion degradation cannot be very fast. The obtained results have revealed that in the case of magnesium alloys, the amorphous microstructure significantly hinders their corrosion in physiological solution. Therefore, it is necessary to pay much attention to the optimization of the chemical composition of magnesium and zinc alloys and the parameters of the melt spinning process to obtain the amorphous microstructure of biomaterials.

## Figures and Tables

**Table 1:** The chemical composition of Ringer’s solution.

Component	Weight (g/dm^3^)
NaCl	8.6
KCl	0.3
CaCl_2_ anhydrous	0.25

**Table 2:** The content of individual alloy components in a given area of Mg_72_Zn_24_Ca_4_ alloy.

Area Number	Mg, % at.	Zn, % at.	Ca, % at.
1	37.2	47.0	15.7
2	96.0	3.9	0.1
3	35.0	47.8	15.0

**Table 3:** The content of individual alloy components in a given area of Zn_87_Mg_9_Ca_4_ alloy.

Area Number	Mg, % at.	Zn, % at.	Ca, % at.	Fe, % at.
1	2.8	88.2	0.1	8.8
2	27.0	72.4	0.2	-
3	3.5	95.3	0.9	-

**Table 4:** Fitting parameters obtained from the EIS measurements of the Mg_72_Zn_24_Ca_4_ alloy immersed in the Ringer solution at 37 °C up to 24 h.

Element	R1 (Ω∙cm^2^)	CPE1-T (F∙cm^−2^∙s^ϕ−1^)	CPE1-P	R2 (Ω∙cm^2^)	R3 (Ω∙cm^2^)	L1 (H∙cm^2^)
Value	22.6	0.0042458	0.65	70.8	169.5	2409
Error	0.13057	5.5056 × 10^−5^	0.0076351	0.69054	7.8144	157.65
Error (%)	0.57	1.30	1.18	0.97	4.61	6.54

**Table 5:** Fitting parameters obtained from the EIS measurements of the Zn_87_Mg_9_Ca_4_ alloy immersed in Ringer’s solution at 37 °C for 24 h.

Element	R1 (Ω∙cm^2^)	R2 (Ω∙cm^2^)	CPE1-T (F∙cm^−2^∙s^ϕ−1^)	CPE1-P	R3 (Ω∙cm^2^)	CPE2-T (F∙cm^−2^∙s^ϕ−1^)	CPE2-P
Value	21.6	14.9	0.00014773	0.88	502.2	0.0063041	0.67
Error	0.073259	0.15484	2.9479 × 10^−6^	0.010437	2.7679	3.3244 × 10^−5^	0.0034085
Error (%)	0.34	1.04	1.99	1.18	0.55	0.53	0.50
